# Conservative Surgery

**Published:** 1864-11

**Authors:** N. W. Abbott

**Affiliations:** Late Surgeon of the 80th Reg’t. Ill. Vols.


ARTICLE XXXVIII.

### CONSERVATIVE SURGERY. CASES OF RESECTION.

REPORTED BY N. W. ABBOTT, late Surgeon of the 80th Reg't Ill. Vols.

At the battle of Mission Ridge, Nov. 24th, 1863, Harrison Hickenlooper, of the 6th Iowa Infantry, fell into my hands for treatment. A minnie ball had passed an inch or so below the head of the humerus; fracturing and very much splintering the shaft of the bone to the extent of more than three inches; extending within the capsular ligament and to the spongy portion of the head of the humerus. Several medical officers who saw the case, concluded that amputation at the shoulder was the only alternative. My observation in other cases of resection led me to hope for favorable results in this. I commenced the operation by a straight incision, some six inches in length, commencing at a point in front and near the acromion process, carrying it downwards along the deltoid muscle on the anterior portion of the joint. The fibres of the deltoid were separated as much as possible with the finger and handle of scalpel. The tendon of the long head of the biceps muscle was found and drawn aside; the splinters of bone were carefully denuded and removed. After denuding the shaft of the humerus to the extent of the fracture, I divided it with a chain saw, through a sound portion; with a bistory I then removed the head of the humerus. The wound was sponged clean and dressed in the usual manner. After the following day this patient passed out of my care and observation, since which time I have not seen him. The following letter will show the extent of his recovery. It was written without solicitation on my part, as I had lost track of him:

"*Albia, Monroe County, Iowa*, April 8th, 1864.

"DEAR SIR:—In accordance with promise, I write to inform you of my recovery from the wound I received at Mission Ridge on the 24th of November last. I was shot, as you will remember, in the right arm, about two inches from the shoulder-joint, and the bone was shattered to pieces. I came to the hospital above Chattanooga, where you were dressing wounds and amputating limbs. I had the good fortune to come under your notice, and you resected the bone of my arm and unjointed it at the shoulder. You took out about four inches of the bone of the arm and dressed it up, and I am happy to inform you that I am now well. It is with the hand of that arm that I am now writing this letter, and to you I am indebted for the preservation of that hand at the present time. I have also pretty good use of the arm, from the elbow to my hand; and I think in time I will have a pretty good arm, although at present it is rather limber, for want of bone."

Again, July 21st, he writes:— * * * "I was glad to hear from you, as I shall always hold you in grateful remembrance. My arm is still gaining in strength and usefulness. I am at present engaged in clerking in a dry-goods store, which business I can do very well. I can also do farm work. I can even chop wood. My arm is about one and a-half inch shorter than the other arm. The elbow-joint is as good as it ever was. I have the same use of my arm, from the elbow down, that I always had. My hand is as strong and as supple as it ever was. I cannot raise my arm from my body outward, without the assistance of the other hand. My arm is shrunk some in size, but I think it will fill out again in time. There is a hard substance, almost as hard as bone, growing from the lower end of the bone that was sawed off, towards the shoulder-joint, and is now two or three inches long, and very solid. The arm is quite well, and I can throw it about any way, violently, without hurting it." * * * * *

(Signed,) "HARRISON HICKENLOOPER,
"*Albia, Monroe County, Iowa.*"

The above letters were written in a fine business hand.

Another case was a Mr. Cox, of the 26th Illinois Infantry, now living in Marion County, Illinois. He was also in the engagement at Mission Ridge, and was wounded in the middle-third of the shaft of the right humerus. The bone was much splintered and comminuted. Near four inches of the bone was involved in the fracture. Splinters of bone were hanging in the opening made by the exit of the ball. Having determined upon the operation of resection, a straight incision was commenced some three inches below the clavicle and over the deltoid muscle, carried downwards along the external border of the biceps muscle, a distance of some six inches. The muscles were pressed aside, and all splinters of bone denuded and removed. The fractured ends of the shaft were carefully denuded, and with a chain saw divided immediately beyond the points of fracture. In this case, also, some four inches of bone was removed. The soft parts were drawn together, and secured by a few sutures and straps; cold water dressings applied. On the following day this patient passed out of my care. I had not seen him, nor heard from him, until he called on me a few days since. I find the arm a-half inch shorter than the other. A new bone is forming from each divided end of the humerus. These new portious of bone are now nearly in contact—a-third or half inch space intervening. This man has not recovered the use of the elbow to any considerable extent. He says, however, it is of late gaining rapidly. He had some abcesses form about the elbow that have delayed his recovery. He doubtless will have a good arm.

At the battle near Dalton, Feb. 25th, 1864, Private Demsey, of the 4th Regular Battery, was wcunded by a piece of shell passing through the upper part of the thigh, crushing in its course the trochanters major and minor, the neck of the femur, and splitting the head of the femur. At the request of Dr. Mensey, Medical Director, I made a resection in this case. I made a straight incision, commencing some two inches *above*, and carried it immediately *behind* the trochanter major, to a point some three inches below; was careful to avoid wounding the sciatic nerve. After removing the splinters of bone, the shaft of the femur was resected with chain saw, one or two inches below the trochanter minor. The head of the femur was then removed with a bistory. This patient was doing well the following day; but at that time he and the medical officer left in charge of him, fell into the hands of the enemy, since which time I have not heard from him.

There are other cases that I will report at some future time, if I can get on track of them and learn results.

